# Diaminated Cellulose Beads as a Sustainable Support for Industrially Relevant Lipases

**DOI:** 10.1021/acssuschemeng.3c07849

**Published:** 2024-05-08

**Authors:** Davide Califano, Rob Schoevaart, Katie E. Barnard, Ciarán Callaghan, Davide Mattia, Karen J. Edler

**Affiliations:** † Naturbeads LTD, 2 Tetbury Hill, Malmesbury SN16 9JW, U.K.; ‡ ChiralVision, 44 Hoog-Harnasch, 2635 DL Den Hoorn, The Netherlands; § Department of Chemical Engineering, University of Bath, Bath BA27AY, U.K.; ∥ Department of Chemistry, University of Bath, Bath BA27AY, U.K.

**Keywords:** cellulose beads, renewable biodegradable carrier, enzyme immobilization, lipases, biocatalysis, biotransformation, mitigation plastic pollution

## Abstract

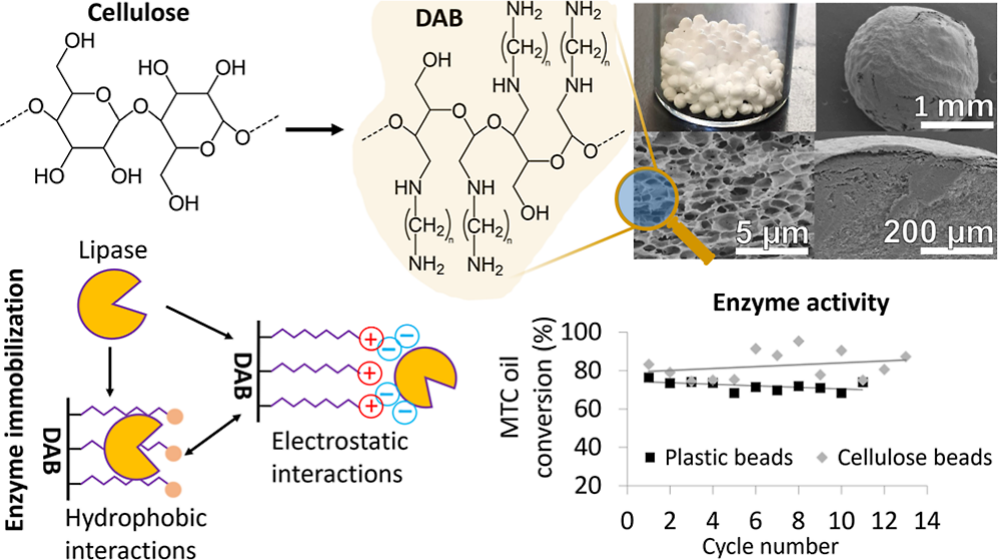

Environmentally persistent polystyrene or polyacrylic beads are used as supports in enzyme large-scale bioprocesses, including conversion glucose isomerization for high-fructose corn syrup production, hydrolysis of lactose, and synthesis of active pharmaceutical ingredients. In this paper, we report the development of a novel sustainable and scalable method to produce diaminated cellulose beads (DAB) as highly efficient alternative supports for industrially relevant lipases. Regenerated cellulose beads were grafted with diaminated aliphatic hydrocarbons via periodate oxidation and reductive amination. The oxidation step indicated that aldehyde content can be easily tuned through the reaction time and concentration of reactants. Reductive amination of dialdehyde cellulose was more efficient as the length of the diaminated hydrocarbon compound increased. Morphological analysis of DAB showed that cellulose chemical grafting enabled the preservation of the bead shape and internal structure upon freeze-drying. Enzymatic degradability studies demonstrated that chemical functionalization did not undermine enzyme cellulose hydrolysis. The addition of aminated moieties on cellulose dramatically increased absorption efficiency for all industrially relevant lipases used, reaching 100% for *Thermomyces lanuginosus* lipase (TLL). Storage and recyclability experiments demonstrated that enzymes were retained and recyclable for at least nine cycles, although the activity gradually declined after each cycle. Medium chain triacylglycerol hydrolysis in a SpinChem reactor using TLL immobilized on 1,6 DAB exhibited higher activity compared to acrylic beads (588 vs 459 U/g) suggesting that biodegradable cellulose-based materials could be a valid and attractive alternative to plastics carriers.

## Abstract 2

Novel cellulose derivatization provides a promising alternative to replace plastics in enzyme immobilization, fostering more sustainable industrial bioprocesses.

## Introduction

The use of immobilized enzymes is increasingly relevant for many large-scale industrial processes including the synthesis of pharmaceuticals, biofuel production, and food biotransformation.^1^ The immobilization of enzymes on recoverable particles is extremely important for the industry as it significantly reduces production costs and improves final product characteristics (purity and safety).^2,3^ Thanks to their low cost and chemical versatility, solid supports based on synthetic polymers such as polystyrene, polyacrylic, and polymethacrylate are widely employed.^4,5^ However, the use of nonbiodegradable enzyme supports presents a significant risk to human health and irreversible damage to the ecosystem because of potential release to the environment.^6^ As such, the use of bioderived polymers is considered a potential alternative to reduce the environmental impact of solid-phase biocatalysis.^7^ Cellulose is an ideal material to be used as an enzyme solid support because of its abundance, low cost, chemical stability, and biodegradability.^8^

Immobilization on solid supports can take place via physical entrapment, hydrophobic/electrostatic interactions, or covalent binding.^2,9^ The immobilization process often involves an adsorption/binding step, in which solid supports are imbibed within an enzyme solution.^10^ High enzyme adsorption efficiency (EAE) is crucial to maximize biocatalytic activity and, thus, the cost-effectiveness of final formulations.^10^ EAE can vary significantly depending on the physicochemical properties of the support used, particularly surface charge, hydrophobicity, and covalent binding sites (or a combination of those).^2^ Native unmodified cellulose does not strongly interact with most proteins, thus a surface functionalization is needed to increase their interactions with enzymes.^11^ Cellulose hydroxyl moieties can be used as chemical anchoring points for the generation of a disparate number of functional groups including carbonyls, amines, hydrocarbon chains, and carboxylic acids.^12^ The use of several types of functionalized cellulose, as support material, for enzyme immobilization has been extensively investigated.^13,14^ However, cellulose and other biopolymers are not economically competitive, when compared with synthetic polymers, due to their higher cost/benefit ratio.^15,16^ To make cellulose commercially appealing for enzyme immobilization, it is necessary to optimize material performance and/or reduce material costs.

Cellulose material optimization can be achieved by improving enzyme loading and activity retention upon recycling through chemical functionalization. In particular, reductive amination proved to be effective at improving biopolymer performance for enzyme immobilization.^17,18^ Reductive amination involves the formation of carbon–nitrogen bonds between a carbonyl and an amino group via an imine intermediate (Schiff base imine).^19^ Enzyme covalent immobilization can be achieved by reductive amination of carbonyls present on support with primary amines on the surface of enzymes.^20^ However, enzyme covalent binding can significantly affect the enzyme 3-D conformation, dramatically reducing its specific activity.^21^ Alternatively, reductive amination can be used to functionalize magnetic nanoparticles, and synthetic-based, and biobased supports by adding aminated groups that increase binding affinity for lipases.^22^ Magnetic carbon nanotubes filled with polyamidoamine dendrimers were used as support to immobilize *Burkholderia cepacia* lipase through covalent binding with a recovered activity of 1.716%.^23^ Bacterial cellulose beads were also used to immobilize Lecitase Ultra enzyme via reductive amination followed by glutaraldehyde cross-linking.^24^

In this work, spherical regenerated cellulose beads (unmodified beads) produced via a dropping method were used as a solid support for biocatalysis. Unmodified beads were functionalized solely through reductive amination with diaminated hydrocarbons for the immobilization of lipase enzymes. The paper explores the effects of chemical grafting with aminated hydrophobic moieties on lipase activity upon recycling and storage. We provide useful insights for further optimization of enzyme immobilization on cellulose supports through hydrophobic and electrostatic interactions as well as structural characterization of functionalized materials. In addition, a comparison between the newly synthesized diaminated cellulose beads (DAB) and the multiton scale produced commercially available polyacrylic bead IB-COV-1 in combination with, for example*, CaLB* (covalent alternative for well-established Novozyme 435) in hydrolyzing medium chain fatty acids (MTC) in a biphasic solvent system using a rotating bed reactor (SpinChem) is presented.

## Results and Discussion

### Diaminated Cellulose Beads Preparation and Characterization

**Figure 1:**
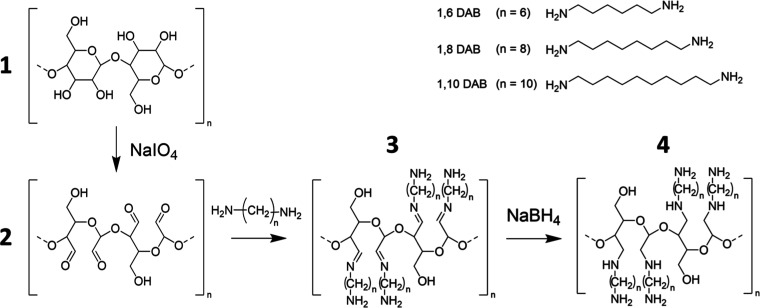
Cellulose bead reductive amination reaction scheme: unmodified beads (1) are oxidized using sodium periodate (NaIO_4_) to produce DAC beads (2). Reductive amination of DAC beads with diaminated aliphatic hydrocarbons (*n* = 6, 8, 10) occurs by forming a Schiff base intermediate (3) and its subsequent reduction with sodium borohydride (NaBH_4_) to produce diaminated cellulose beads (DAB) (4).

Diaminated cellulose beads (DAB) were prepared through a multistep functionalization process using regenerated cellulose beads (unmodified beads) as a starting material. Unmodified beads were first converted into dialdehyde cellulose (DAC) beads and then grafted with diaminated aliphatic hydrocarbons (1,6-diaminohexane, 1,8-diaminooctane, and 1,10-diaminodecane) via reductive amination (Figure 1).

**Figure 2:**
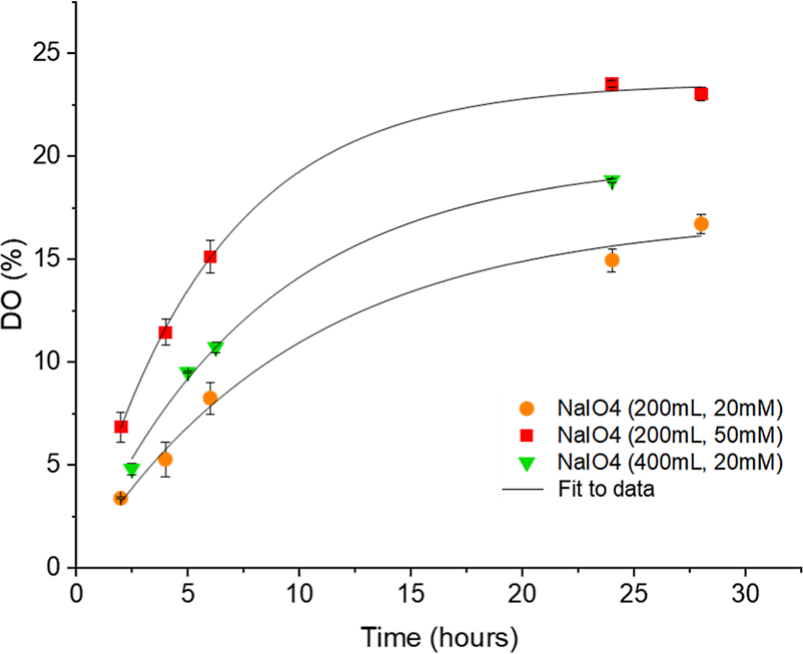
Periodate oxidation reaction kinetics: cellulose DO was determined by acid–base titration. The DO linearly increases in the first 6 h of reaction, after which it stabilizes, reaching a plateau after 24 h. Lines represent the curve fitting using eq 1. Bars indicate standard deviation resulting from withdrawn aliquots (*n* = 3). Tabulated DO values and standard deviations are in Table S1.

The resulting DAB maintained their initial spheroidal shape and size with an average diameter of 1.92 ± 0.18 mm (1.98 ± 019 mm before chemical functionalization), while the particle color changed from translucent white to pale yellow (Figure S1). Periodate oxidation of cellulose beads was used to produce aldehyde moieties through vicinal diol oxidation.^25^ The oxidation kinetic curves showed a linear trend in the first 6 h of reaction, after which the degree of oxidation (DO) attenuated, reaching a plateau due to reactant depletion (Figure 2).

All reactions showed pseudo-first-order kinetics, as expected for systems in which the concentration of one of the reactants (cellulose) is in excess, while the second reactant (NaIO_4_) is consumed. Kinetic results were fitted with eq 1, where DO represents the degree of oxidation and *t* represents the time in hours. By fitting periodate oxidation kinetic curves, it is possible to determine the maximum DO and the initial slope, which correspond, respectively, to the values of coefficients *a* and *b* (Table 1).
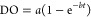
1

**Table 1:** Reaction Conditions, and Equation Coefficient *a* and Pseudo-First-Order Constant *b*, Obtained by Fitting Data from Periodate Reaction Kinetics Measured with a Pseudo-First Order Model (eq 1)

sample	NaIO_4_ (V, molarity)	NaIO_4_ mass (mmol)	equation coefficient	pseudo-first order constant
			*a*	*b t* ^–1^
NaIO_4_-10	200 mL, 50 mM	10	23.74 ± 0.20	0.170 *t* ^–1^ ± 0.005
NaIO_4_-8	400 mL, 20 mM	8	19.88 ± 0.60	0.124 *t* ^–1^ ± 0.008
NaIO_4_-4	200 mL, 20 mM	4	17.12 ± 0.76	0.102 *t* ^–1^ ± 0.012

The concentration of NaIO_4_ is the major factor influencing the initial rate of reaction, while the maximum DO of cellulose is dependent on both the concentration and absolute amount of NaIO_4_.

**Figure 3:**
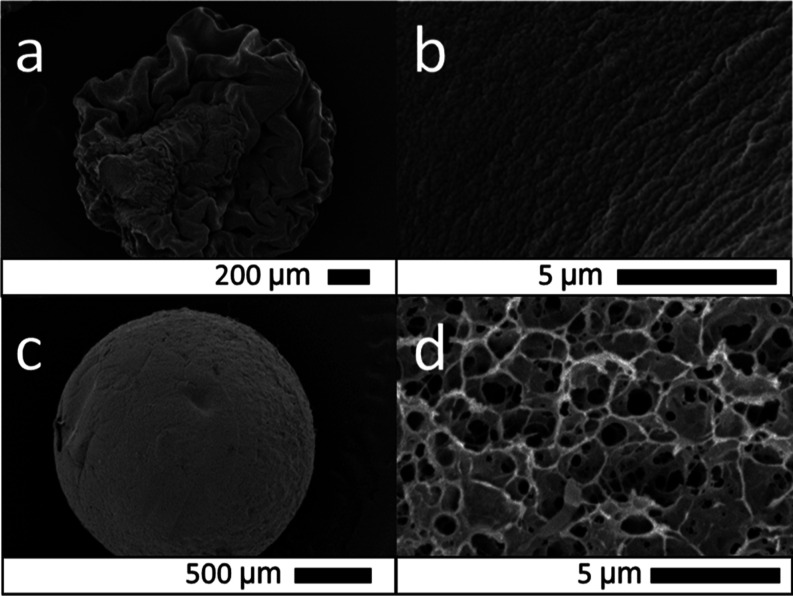
Scanning electron micrographs of (a,b) unmodified beads and (c,d) DAC beads that have undergone periodate oxidation.

Scanning electron microscopy analysis showed that DAC beads retained the shape and porosity of the starting beads upon freeze-drying (Figure 3c,d), whereas the latter exhibited the formation of wrinkles and reduced porosity that might be associated with cellulose hornification (Figure 3a,b).^26^ This difference could be attributed to the increase in amorphous cellulose in the beads after periodate oxidation, caused by the partial removal of hydroxyl groups (responsible for the formation of hydrogen bonding). As previously demonstrated, periodate oxidation causes structural changes in cellulose polymer self-assembly thanks to the reduction of highly packed crystalline regions.^20,27^ Substitution of hydroxyl groups with aldehydes and the opening of glucopyranose ring makes cellulose more flexible and less capable of forming ordered structures.^28^

**Figure 4:**
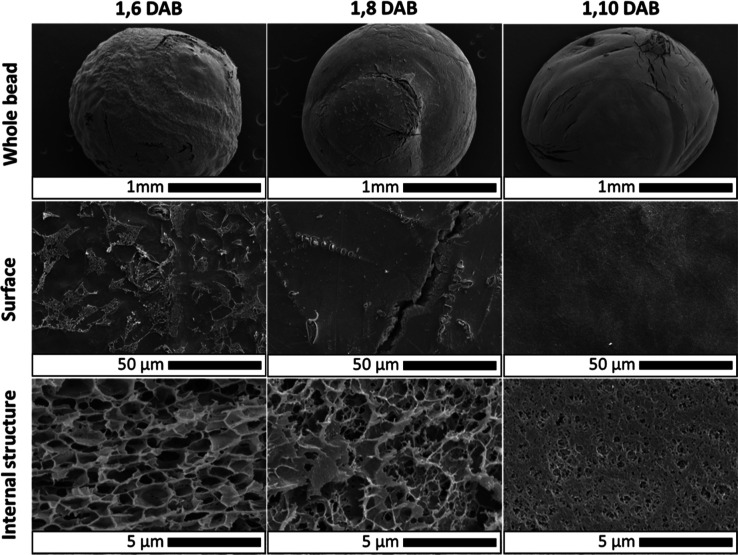
Scanning electron micrographs of (top row) diaminated beads, (middle row) surface, and (bottom row) cross-section upon freeze-drying as a function of grafted hydrocarbon diamine chain length.

Since cellulose with a higher DO tends to lose its mechanical stability,^20^ only DAC beads with a DO of 11 ± 0.63% were chosen as a starting material for the subsequent reductive amination reaction. Reductive amination was performed using three diaminated hydrocarbon compounds differing in their chain length (6, 8, and 10 carbons). Increasing the hydrocarbon chain length in the reductive amination step resulted in a reduction of the internal porosity of and smoother surfaces for DAB (Figure 4).

Although the mechanism behind these morphological changes at the mesoscale is not entirely clear, it is plausible to assume that cellulose surface hydrophobicity plays an important role. In fact, it has been shown that cellulose hydrophobic functionalization (with methyltrimethoxysilane) also induces a variation in porosity resulting in the formation of aerogels upon freeze-drying.^29,30^

**Figure 5:**
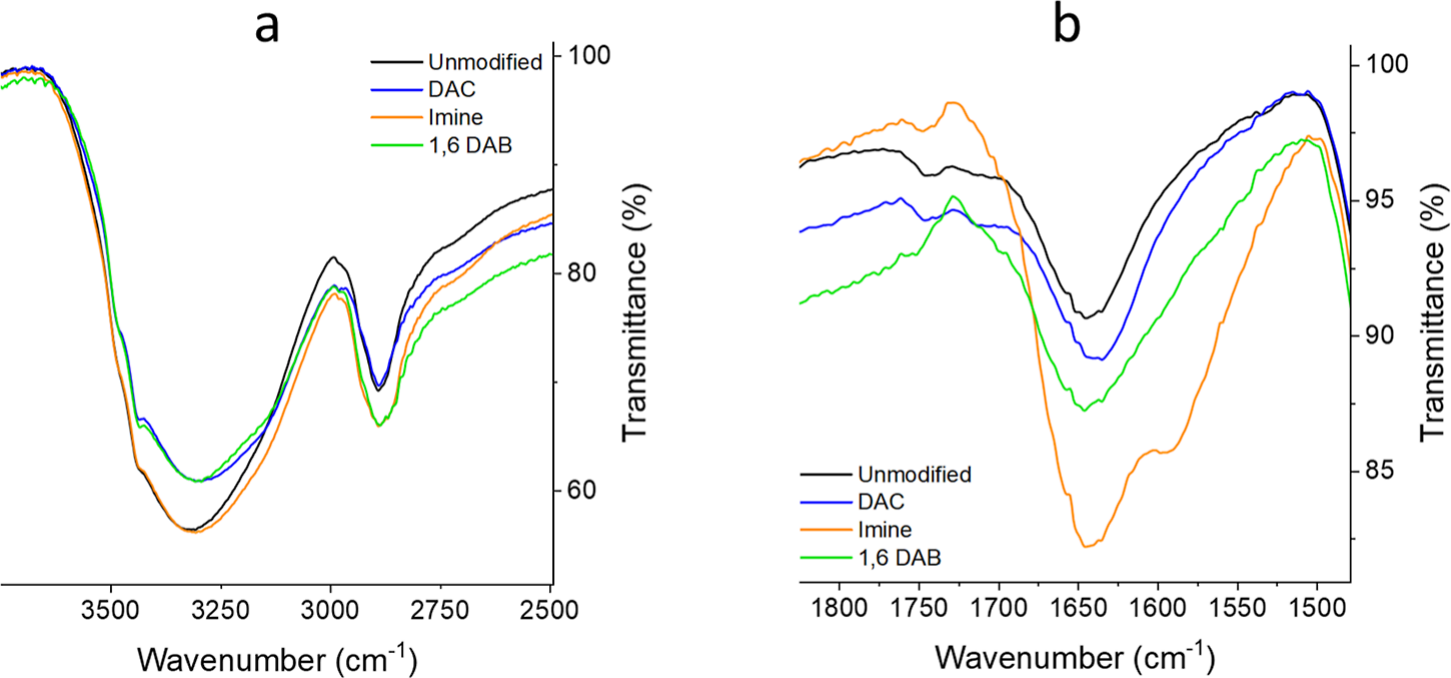
FTIR spectra of cellulose powders obtained from beads at each stage of chemical grafting through reductive amination. Unmodified cellulose and DAC (black line and blue line, respectively) show a lower absorption in the region between 1500 and 1700 cm^–1^ compared to the imine reaction intermediate and 1,6 DAB (orange and green line, respectively) indicating the occurrence of reductive amination (b). The region between 2500 and 3750 cm^–1^ highlighted the presence of intramolecular/intermolecular O–H stretching and cellulose/water hydrogen bonding (a).

The functionalization of the cellulose via reductive amination was confirmed through FTIR analysis of DAC beads functionalized with 1,6-diaminohexane (1,6 DAB). The DAC infrared spectrum (Figure 5a) exhibited a slight increase in intensity at 1713 and 1745 cm^–1^ which can be attributed to the presence of aldehyde groups (C=O stretching).^31^ However, a peak in the same region (1745 cm^–1^) was also observed in the unmodified cellulose, making direct differentiation between unmodified and DAC samples difficult. This could be explained by the formation of hydrated carbonyl species present in the unmodified cellulose, such as hemiacetal and hemialdol, which adsorb in the same region.^32,33^ The Schiff base intermediate (imine) was characterized by the presence of a distinct increased absorption in the region between 1580 and 1690 cm^–1^ (Figure 5b). Infrared absorption in this region can be associated with the presence of imine/oxime (1640–1690 cm^–1^, C=N stretching) and amine (1650–1580 cm^–1^, N–H bending) both confirming the grafting of cellulose with the diaminated compounds.^34,35^ After the reduction step and washing, the Schiff base intermediate showed a significant reduction of C=N stretching and N–H bending absorption. However, the absorption intensity between 1580 and 1690 cm^–1^ was still higher than the absorption of cellulose prior to the reductive amination reaction, confirming the successful reduction of the imine and, thus, the presence of diaminated moieties on the cellulose (Figure 5b). Cellulose functionalization was further confirmed by the increase of the alkane C–H stretching (2840–3000 cm^–1^) in the imine and 1,6 DAB samples (Figure 5a).^34^ Differences in absorption in the region between 3000 and 3500 cm^–1^ can be attributed to the variation of intramolecular/intermolecular, and cellulose/water hydrogen bonding (O–H stretching).^36^ The unmodified cellulose presents a higher absorption when compared to DAC and 1,6 DAB, suggesting the elimination of hydroxyl groups caused by periodate oxidation of cellulose.^37^ Also, the addition of hydrophobic aliphatic moieties (1,6-diaminohexane) could be responsible for decreasing the presence of solvated water on the cellulose surface.

The ninhydrin assay provides additional validation of the presence of amine groups, both primary and secondary amines, grafted onto cellulose following the beads’ amination reaction. Although the ninhydrin assay is primarily employed for amino acid/protein quantification,^38^ it has also been extensively utilized for surface characterization of colloidal and noncolloidal particles.^39−41^ Upon reaction with ninhydrin, the reaction mixtures exhibited an evident contrast between unmodified and DAB samples. Unmodified beads resulted in a subtle alteration in color intensity within the ninhydrin solution, whereas DAB-modified beads provoked a dramatic change of color toward a deep purple hue. Amine quantification was achieved through linear regression analysis of a calibration curve utilizing glutamic acid as a standard. Interestingly, the amine content was significantly higher in 1,10 DAB (261.58 ± 25.81 μmol/g) and 1,8 DAB (234.35 ± 33.39 μmol/g) when compared to 1,6 DAB (170.91 ± 17.00 μmol/g), suggesting a potential increase in the efficiency of the amination reaction as hydrocarbon chain length increased (Table 2).

**Table 2:** DAB Amine Concentration, Following the Animation Reaction Was Determined Using the Ninhydrin Assay^a^

sample	1,6 DAB	1,8 DAB	1,10 DAB
amine concentration (μmol/g)	170.91 ± 17.00	234.35 ± 33.39	261.58 ± 25.81

a The table presents the amine concentration values in micromoles of amine per gram of dry weight cellulose. Errors represent the standard deviation (*n* = 3).

### DAB Enzyme Degradability

**Figure 6:**
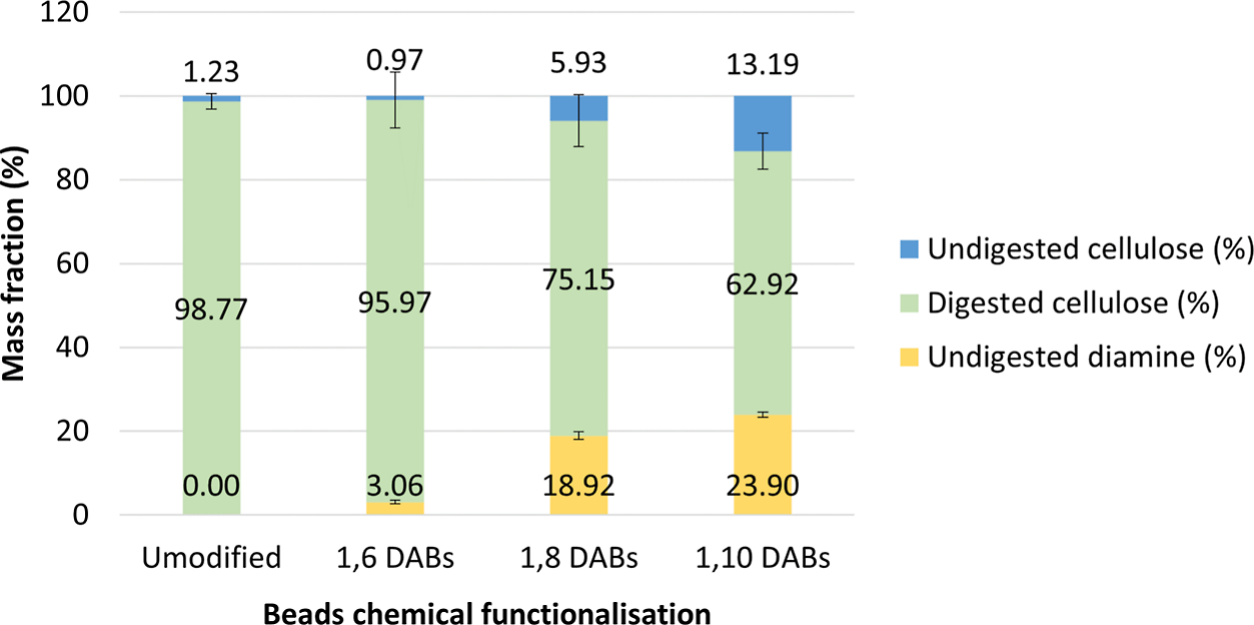
Cellulose bead enzymatic digestion with cellulases. The introduction of hydrophobic aminated moieties affects the action of cellulases, decreasing the mass fraction of digestible cellulose.

The degradability of DAB was tested in vitro using a fungal cellulose-lytic enzyme cocktail (from *Trichoderma reesei*). The total hydrolyzed mass reached 95.97 ± 6.72% and 75.15 ± 6.21% for reactions containing 1,6 and 1,8 DAB, respectively, while it reached 62.1 ± 4.25% in 1,10 DAB (Figure 6—green bars). The cellulose fraction in 1,6 and 1,8 DAB was almost entirely digested after 22.5 h of incubation, suggesting the grafting reaction did not significantly affect the hydrolysis of β-1,4 glycosidic bonding. The cellulose undigested fraction (Figure 6—blue bars) significantly increased in 1,10 DAB, suggesting that activity of lytic enzymes was inhibited by the presence of 1,10-diaminodecane moieties. The molar fraction of diamines in DAB (0.026, 0.131, and 0.139 mol/mol for 1,6 DAB, 1,8 DAB, and 1,10 DAB respectively) was directly proportional to the length of the hydrocarbon chains (Figure 6—yellow bars). However, the diamine/cellulose molar fraction did not increase linearly concerning the diamine hydrocarbon length suggesting that a higher degree of substitution was reached with more hydrophobic diamines. This phenomenon could be explained by the high affinity of cellulose toward hydrophobic species, as also observed for cellulose binding domain proteins, and polyphenols.^42−44^

#### Enzyme Immobilization on DAB

**Figure 7:**
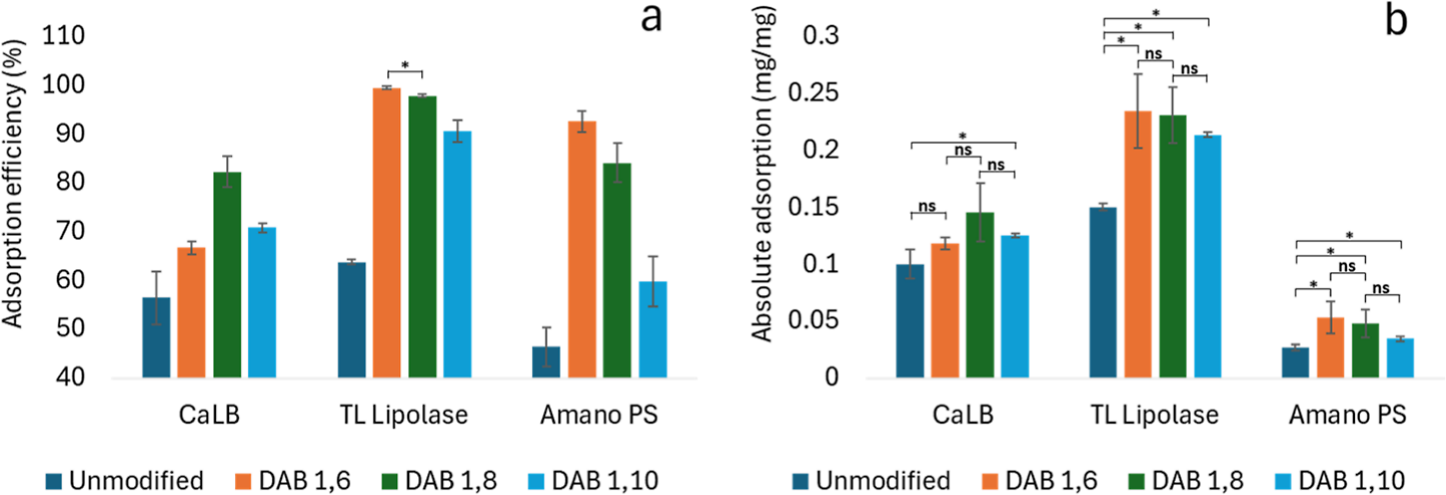
(a) Adsorption efficiency and (b) absolute adsorption of CaLB, TLL, and Amano PS enzymes on DAB. Error bars represent the standard deviation (*n* = 3), “ns” stands for nonsignificant differences, and “*” stands for *p* value < 0.05 (one-way analysis of variance test).

Three different lipases were used to evaluate the immobilization efficiency of DAB, namely, *Candida albicans* Lipase B (CaLB), Lipase from *Thermomyces lanuginosus* lipase (TLL) and Amano Lipase from *Candida antartica* (Amano PS). Cellulose amination significantly improved the overall immobilization performance in terms of adsorption, activity retention, and storage. Enzyme adsorption was significantly higher in all DAB samples (Figure 7), indicating that chemical grafting improved the cellulose binding affinity for enzymes. The 1,6 DAB exhibited the best adsorption efficiency for TLL and Amano PS, while CaLB enzyme was adsorbed more efficiently by 1,8 DAB. The length of the hydrocarbon chain in DAB influenced the enzyme adsorption, but no general trend could be identified for all enzymes. For TLL and Amano PS, adsorption efficiency seems to get worse as the hydrocarbon chain length increases, while for CaLB the longest diamine hydrocarbon (1,8 DAB) showed the best results (Figure 7a). The differences in adsorption performance could be determined by a multitude of factors, including mass fraction of diaminated moieties, cellulose surface charge, and hydrophobicity.

**Figure 8:**
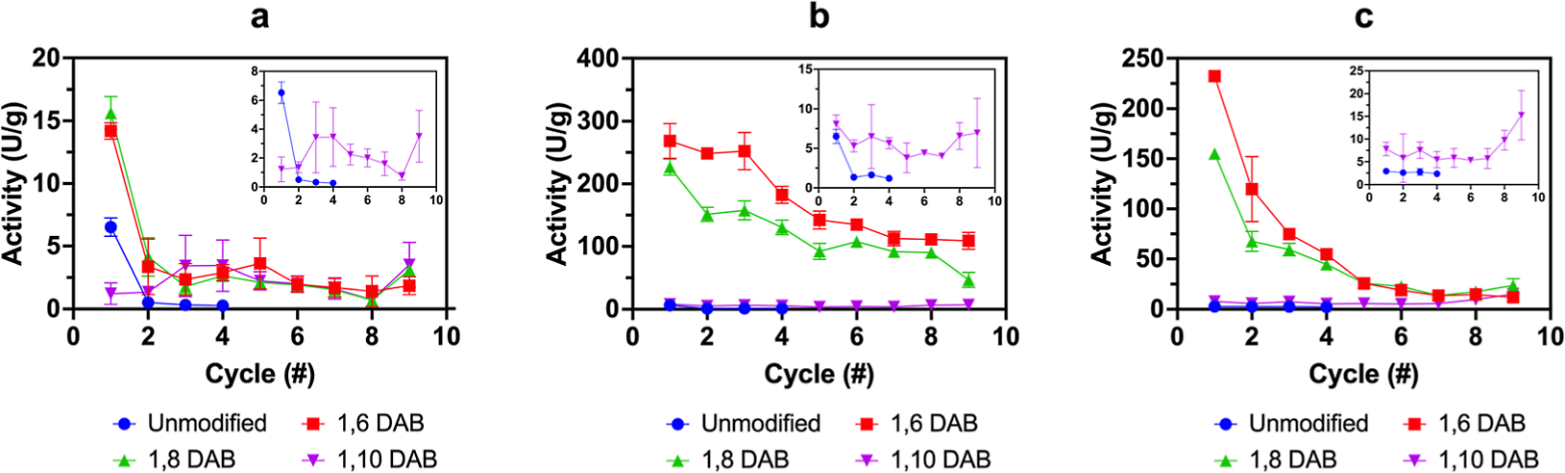
Activity (hydrolysis of *p*-nitro phenyl butyrate) retention upon recycling of immobilized enzymes, (a) CaLB, (b) TLL, and (c) Amano PS on 1,6 DAB, 1,8 DAB, 1,10 DAB, and unmodified beads. The enzyme activity retention was significantly higher for 1,6 and 1,8 DAB when compared to that for 1,8 DAB and unmodified cellulose beads. Insets show magnified sections of samples with lower activities.

To assess the lipase enzyme activity upon immobilization, a standard assay consisting of the hydrolysis of *p*-nitro phenyl butyrate (pH ∼ 7.4, at 25 °C) in an aqueous environment was used. The activity of all immobilized lipases was significantly enhanced by the introduction of diaminated moieties on cellulose. The enzymes showed different activity retention upon recycling (Figure 8) indicating that the physicochemical properties of the immobilized system can influence enzyme catalytical activity. The activity of immobilized CaLB dramatically dropped after the first cycle (Figure 8a), while the activity of TLL and Amano PS was retained over a higher number of cycles (Figure 8b,c). Immobilized TLL and Amano PS exhibited a different recyclability profile with the best performance observed in TLL immobilized on 1,6 DAB and 1,8 DAB hydrolysis of *p*-nitro phenyl butyrate was measured each 24 h for 9 cycles. In 1,6 DAB and 1,8 DAB, 40% of immobilized TLL activity was retained suggesting that partial enzyme deactivation or leaching occurred upon recycling the beads. The activity of CaLB immobilized on 1,6 DAB and 1,8 DAB sharply dropped after the first cycle while CaLB activity on 1,10 DAB remained stable around 4 U/g. The recycling performance of enzyme/DAB hybrids is dependent on the type of enzyme used, suggesting that protein structural features are vitally important to retain and preserve enzyme activity.^45^

Hydrocarbon chain length strongly affected the *p*-nitro phenyl butyrate hydrolysis performance of the immobilized enzymes. The 1,6 DAB exhibited the highest activity, suggesting that a lower cellulose surface hydrophobicity can positively affect lipase triglyceride hydrolysis in an aqueous environment.^46^ Although hydrophobic regions could facilitate the lipase binding onto cellulose via a hydrophobic interaction, an excessive surface hydrophobicity could reduce the amount of water in the proximity of enzymes, thus reducing activity in aqueous environments. In fact, the activity of all lipases (for hydrolysis of *p*-nitro phenyl butyrate in an aqueous environment) decreases as the length of hydrocarbon chain increases. This reduction in enzyme activity is likely to be caused by a reduced accessibility of both water and the *p*-nitro phenyl butyrate substrate on the cellulose surface when a more hydrophobic moiety is present. To confirm this hypothesis, two parallel activity tests of immobilized TLL were performed: (1) direct esterification of propyl-laurate conducted in an organic environment; (2) hydrolysis of tributyrin (TBU) in an aqueous environment. Direct esterification experiments showed no differences in activity in relation to hydrocarbon chain length since this reaction does not require water, while TBU hydrolysis in an aqueous environment dramatically decreased for the longer aminated chains (Figure S2) confirming the negative effect of reduced local water concentrations when lipase is immobilized on more hydrophobic carriers.

#### Demonstration Reaction of Immobilized Enzyme in a Rotating Bed Reactor

**Figure 9:**
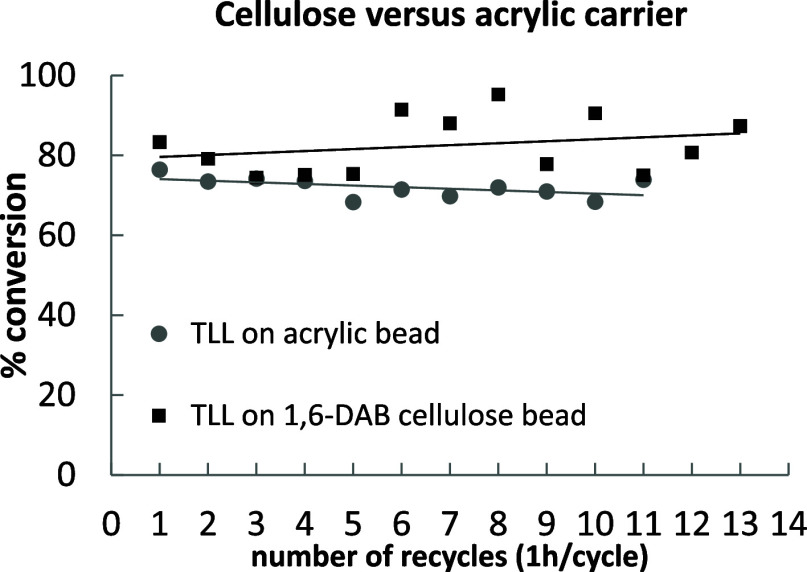
Activity of TLL immobilized on acrylic beads (circles, gray line) or on 1,6-DAB cellulose beads (squares, black line) in MCT oil hydrolysis in a SpinChem rotating bed reactor at 40 °C. Each cycle lasts 1 h.

To investigate the potential for scaling-up the use of enzymes supported on DAB for biocatalysis applications, a two-phase reaction system was used in a rotating bed reactor (SpinChem). In a direct comparison test, an equal amount of lipase from TLL was immobilized on commercial acrylic beads (IB-COV-1) (covalent binding) and cellulose beads (1,6-DAB) (ionic binding and physical adsorption) and tested for medium chain triglycerides (MCT) oil hydrolysis. Each cycle was run for 1 h, after which about 70–90% conversion was reached in each case. About five cycles were run in a day, and after the last cycle the enzyme was left in the reaction mixture overnight without heating or stirring. After 12 cycles the lipase remained stable in the cellulose beads and reached higher conversion and activity (87%, and 588 U/g respectively) than the same enzyme immobilized on conventional acrylic beads (68%, and 459 U/g respectively) (Figure 9).

Adsorbed or ionically bound enzymes in two-phase reaction systems or systems with a certain percentage of cosolvent are known to leach off the carrier resulting in quick activity loss in recycles and contamination of the product with protein.^47^ In the MCT oil hydrolysis test, it was clearly demonstrated that the TLL strongly binds to the grafted cellulose in 1,6-DAB beads and that no desorption takes place in water—C8–C10 MCT oil mixtures. Contrary to what we observed during the hydrolysis of *p*-nitro phenyl butyrate experiment (Figure 8), where the activity loss over 9 catalytical cycles for TLL immobilized on 1,6 DAB is ≈55% (Figure S4). This dramatic change in performance could be explained by the difference in reaction conditions used (substrate, time span between cycles, temperature, experiment output, and one phase vs two phases system). Avoiding a covalent linkage results in higher activity recovery during immobilization. Ionically/physically adsorbed enzymes have more conformational freedom and hence a higher activity when compared to other systems based on covalent binding.^48^

## Conclusions

The alarming presence of microplastics in the environment poses a threat to human health and ecosystems. As microplastics are impossible to separate and/or recycle on a large scale, the development of novel biodegradable and sustainable substitutes constitutes the only solution to mitigate their accumulation in the environment. Here, a diaminated cellulose-based carrier to be used for the immobilization of commercial lipases was designed and tested. Diaminated cellulose carriers were successfully synthesized by using reductive amination chemistry on regenerated DAC beads. The introduction of aminated hydrocarbons on the cellulose surface through reductive amination improved enzyme immobilization efficiency and activity retention upon recycling. The presence and length of grafted aminated hydrocarbons affected the shape and porosity of the beads but did not affect cellulose enzyme degradability. The increased length of aminated hydrocarbons had a negative effect on the activity of immobilized lipases in an aqueous environment, while activity in organic solvent remained unvaried. The best combination of enzyme/carrier type was found to be TLL immobilized on 1,6 DAB which reached an adsorption efficiency of 100% and a maximum activity of 270 U/g of dry carrier. This formulation outperformed TLL immobilized covalently on a conventional nonrenewable acrylic epoxide carrier in a two-phase reaction system and showed no loss of activity even after extensive reuse (12 cycles). Considering the current reliance of industry on acrylic-based beads as a support, the cellulose-based technology herein presented represents an appealing opportunity to make large-scale bioprocesses more sustainable.

## Materials and Methods

### Materials

Methanol (VWR Chemicals, ≥99.8), hydroxylamine hydrochloride (159417, Sigma-Aldrich, ≥99%), sodium hydroxide (1.06498, Supelco, ≥99.0%), *p*-nitrophenyl butyrate (N9876, Sigma-Aldrich, ≥98%), TBU (W222322, Sigma-Aldrich), lauric acid (8.05333, Sigma-Aldrich), 1-propanol (1.00996, Sigma-Aldrich), MCT-oil (Brenntag). Lipase enzyme formulations *C. antartica* lipase B (lipozyme, CALB L), *T. lanuginosus* lipase (Lipolase 100 L) were obtained from Novozymes A/S (Bagsværd—Denmark). *Pseudomonas cepacia* lipase (Lipase PS) was obtained from Amano Enzyme Europe Ltd. (Milton—United Kingdom). 1,6-Diaminohexane (8.04323, Sigma-Aldrich, ≥98%), 1,8-diaminooctane (D22401, Sigma-Aldrich, ≥98%), 1,10-diaminodecane (D14204, Sigma-Aldrich, ≥97%), bovine serum albumin (05470, Sigma-Aldrich, ≥96%), bicinchoninic acid (BCA) solution (B9643, Sigma-Aldrich), Bradford reagent (B6916, Supelco), d-(+)-glucose (G8270, Sigma-Aldrich, ≥99,5%), microcrystalline cellulose (MCC) (435236, Sigma-Aldrich, LOT #MKCF1486), sodium borohydride (MFCD00003518, Acros Organics, ≥98%), sodium periodate (MFCD00003534, Acros Organics, ≥98,8%), Ninhydrin Reagent (N7285, Sigma-Aldrich, 2% w/v solution), Glacial acetic acid (695092, Sigma-Aldrich, ≥99.7%), l-glutamic acid (G1251, Sigma-Aldrich, ≥99%), phosphate saline buffer (P4417—Sigma-Aldrich), sodium acetate (S2889 Sigma-Aldrich, ≥99%), sodium phosphate dibasic (S9763, Sigma-Aldrich, ≥99%), 1-ethyl-3-methylimidazolium acetate [EMIm][OAc] (Proionic, purum ≥ 98%), Cellulase from *T. reesei* (C2730—Sigma-Aldrich), 3,5-dinitrosalicylic acid (D0550, Sigma-Aldrich, ≥98%), were all used as received unless otherwise stated.

### Preparation of Regenerated Cellulose Beads

#### Cellulose Dissolution and Beads Regeneration

**Figure 10:**
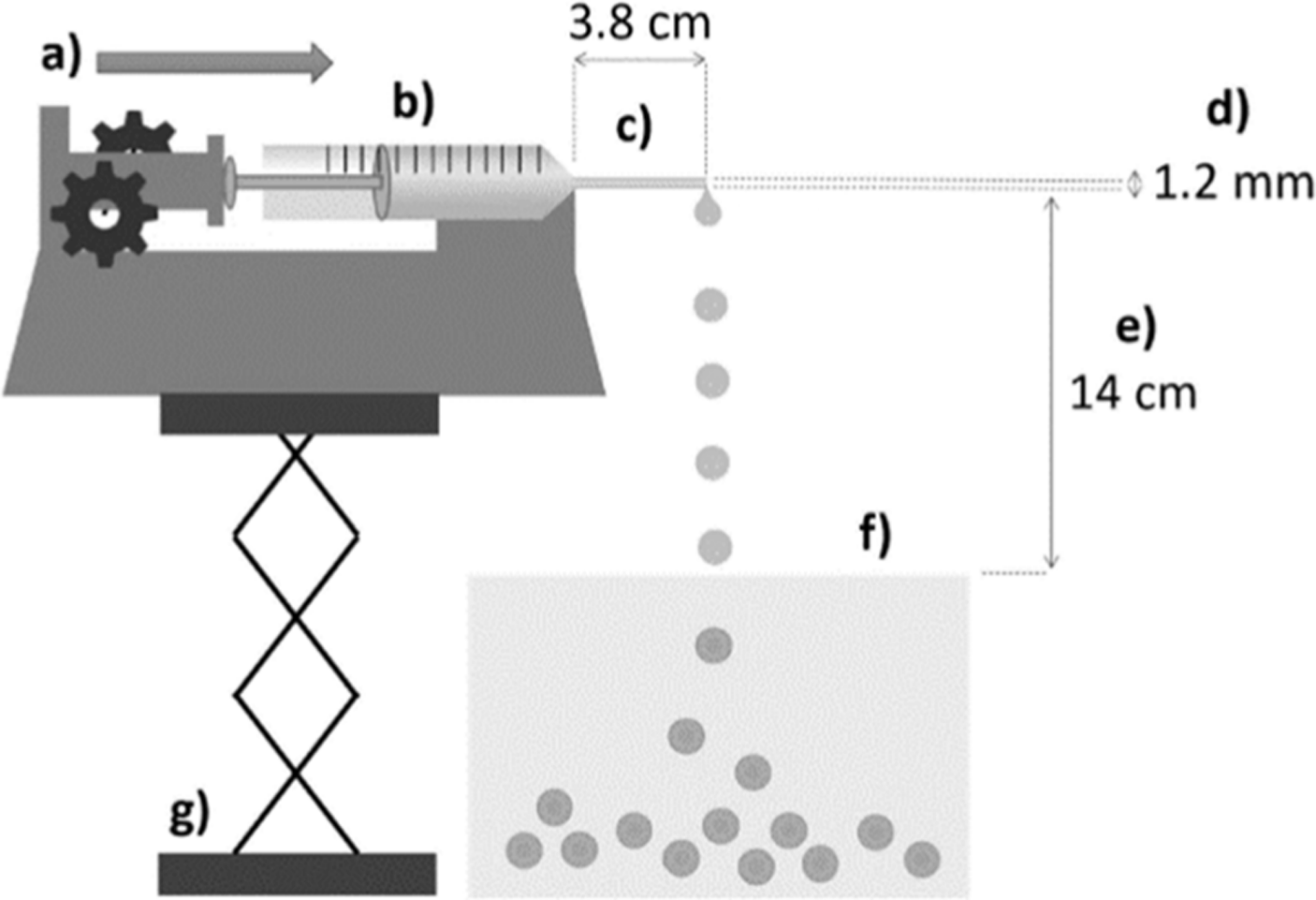
Dropping system used for the synthesis of regenerated cellulose beads, used as a starting material for the preparation of DAB.

To prepare 500 g of 6 wt % cellulose solution, 30 g of MCC was dispersed in 40 g of water with an overhead stirrer (900 rpm) at room temperature. Then, 430 g of 1-ethyl-3-methylimidazolium acetate ([EMIm][OAc]) were added dropwise into the dispersion, and the mixture was stirred for 4 h until all cellulose powder was dissolved. Cellulose beads were prepared via the regeneration of cellulose droplets into a deionized (DI) water bath (antisolvent). The cellulose solution was dropped and precipitated from a 1.2 mm × 38 mm stainless steel needle into DI water using a syringe pump (KdScientific −210) set to a constant flow rate, such that individual droplets formed (Figure 10). The dropping height was set at 14 cm from the surface of the water in the precipitation bath. Beads purification was achieved by soaking the beads in abundant DI water and sieving with a stainless-steel sieve at least four times (each wash with 10 volumes of water with respect to the bead’s volume).

### DAB Synthesis

#### Cellulose Bead Oxidation (to Form Dialdehyde Cellulose Beads)

The cellulose beads (100 g beads) were suspended in 400 or 200 mL of sodium periodate (NaIO_4_) at a concentration of 50 or 20 mM and reacted at 25 °C for 28 h under mild agitation using an overhead stirrer to form DAC beads, and aliquots of beads were withdrawn at 2, 4, 8, 24, and 28 h to calculate kinetics. Excess NaIO_4_ was removed by washing the beads (soaking and sieving with a 1 mm mesh-size stainless-steel sieve) in abundant DI water until the absorption of the supernatant at 290 nm was zero (periodate adsorption peak). The dialdehyde beads (DAC beads) were stored in DI water at room temperature. The DO was determined via acid–base titration.^20^ 2 g of oxidized beads were homogenized with 3 mL of DI water using an Ika Ultra-Turrax T8 and dispersed into 25 mL of a 0.25 M hydroxylamine hydrochloride solution (adjusted with 0.1 M NaOH to pH 4). The HCl released in the reaction between the aldehydes and hydroxylamine hydrochloride was titrated against 0.1 M NaOH using an Accumet pH meter (Fisher Scientific), and equivalent point peaks were obtained from the first order derivative of pH changes against volume added (dpH/dV).

#### Cellulose Bead Amination

DAC beads were solvent exchanged (from water to methanol) by washing beads two times with four volumes of methanol (with respect to bead volume). The beads (20 g) were then transferred into a 50-mL falcon tube with an extra 25 mL of methanol. 1,6-Diaminohexane, 1,8-diaminooctane and 1,10-diaminodecane were dissolved into the methanol/bead mixture. The amount of diamine compounds corresponded to 1.2 equiv with respect to the carbonyls present in DAC beads in the tube. The reaction was conducted at room temperature for 24 h, after which beads were sieved and immersed into a solution of NaBH_4_ in methanol containing 1.5 equiv of NaBH_4_ with respect to the in respect of carbonyls on the cellulose beads (imine reduction step). After the reduction step, the beads were washed with DI water thoroughly to remove methanol and NaBH_4_. The presence of amines following beads amination was evaluated by reacting them with ninhydrin. To perform the ninhydrin test, approximately 0.5 mg of dry unmodified (negative control) and DAB beads were homogenized in 2 mL of DI water using an Ika Ultra-Turrax T8. Beads dispersions (2 mL) were then combined with 1 mL of ninhydrin reagent and subjected to a 10 min reaction in a boiling water bath. After the reaction, the mixtures were cooled to room temperature, and 5 mL of 95% ethanol was added to each tube. The resulting mixtures were centrifuged to eliminate any solids, and a supernatant aliquot was taken for absorbance measurement at 570 nm. Amine concentration values were determined by extrapolating from a calibration curve generated using known concentrations of the amino acid aspartic acid via linear regression. FTIR analysis was performed using a PerkinElmer Frontier-FTIR P with a universal Miracle ATR sampling accessory. All samples were oven-dried for 1 day at ∼60 °C before analysis. All FTIR spectra were obtained with 32 scans acquired in the range 4000–450 cm^–1^.

### Bead Morphological Characterization

#### Beads Size

Brightfield images of beads were obtained with a stereoscopic microscope (Motic—SMZ143). Bead sizes were determined through image analysis using ImageJ software.^49^

#### Scanning Electron Microscopy

Scanning electron micrographs were produced by using a JEOL SEM648OLV microscope. The beads were flash frozen in liquid nitrogen and lyophilized using a MiniLyotrap instrument (LTE scientific). To prepare cross sections, single wet beads were cut with a sharp blade prior to flash freezing and lyophilization. Before imaging, whole beads and cross sections were coated with gold for 5 min to form a 10 nm layer (Edwards sputter coater, S150B).

### Enzyme Adsorption Step

#### Enzyme Adsorption Efficiency

To evaluate adsorption efficiency, 0.5 g of hydrated DAB was incubated with 1 mL of lipase enzyme solutions for 16 h at room temperature. Lipase enzyme formulations namely CaLB, TLL, and *P. cepacia* lipase (Amano PS) were diluted 4 times with DI water (0.25 V/V %). The concentrations for each lipase after dilution were the following: 8.86 ± 0.32 mg/mL for CaLB, 15.19 ± 1.18 mg/mL for TLL, and 2.87 ± 0.05 mg/mL for Amano PS. Enzyme concentration was measured using the Bradford method^50^ or with BCA coloring. Bradford reagent (195 μL) was transferred to a 96 well-plate, mixed with enzyme solutions between 0.1 and 1.4 mg/mL (5 μL) and incubated for 10 min prior to a spectrophotometric reading (at 595 nm) (FLUOstar Omega Microplate Reader—BMG LABTECH). BCA reagent (1000 μL) was added to diluted enzyme solutions between 0.01 and 1 mg/mL (300 μL) and incubated for 30 min at 40 °C before transferring to a 1.5 mL cuvette followed by spectrophotometric reading (at 562 nm). Calibration curves were obtained using known concentrations of BSA determined via UV absorption at 280 nm (molar extinction coefficient and molecular weight at 43,824 M^–1^ cm^–1^ and 66430.3 Da, respectively). EAE was determined as the mass fraction migrated from the enzyme solution into DAB by comparing the enzyme concentration before (*iC*) and after (*fC*) incubation with DAB (eq 2). A ANOVA test was performed to assess the significant variations in enzyme adsorption among various DAB materials.
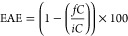
2

#### Enzyme Activity Retention upon Recycling and Storage

Beads containing enzymes, obtained from adsorption experiments, were employed for all activity tests. Enzyme activity was determined in vitro by measuring the conversion over time of *p*-nitrophenyl butyrate to *p*-nitrophenol and butyric acid by UV–vis spectrophotometry. About 0.5 mg of enzyme-functionalized DAB (dry weight) was suspended in 2 mL of phosphate buffer saline (PBS, pH ∼ 7.4) containing 1 mM of *p*-nitrophenyl butyrate. Reaction progress was monitored continuously by UV–vis spectrophotometry (FLUOstar Omega Microplate Reader, BMG LABTECH) at 25 °C by measuring the absorbance at 400 nm for approximately 5 min. One unit (*U*) of activity corresponds to the release of 1 micromole of *p*-nitrophenol per minute at pH 7.4 and 25 °C using *p*-nitrophenyl butyrate as substrate. Activity was reported as units (*U*) of activity per dry weight of functionalized beads (*U*/*g*) according to eq 3.
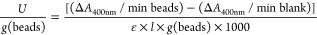
3where ε is the molar extinction coefficient of *p*-nitrophenol (0.01725 μM^–1^ cm^–1^), Δ*A*_400nm_ is the absorbance difference (final absorbance subtracted by the initial absorbance), path length (*l*) = 1 cm and *g*(beads) of the beads dry weight in grams. To measure activity upon recycling, enzyme-functionalized DAB was reused once a day and stored at 4 °C in fresh PBS overnight. Prior to each enzyme activity measurement, enzyme-functionalized DAB was washed with fresh PBS at room temperature. Enzyme stability upon storage was determined by measuring activity retention of enzyme functionalized DAB after 4 months of storage in DI water at 4 °C.

#### Tributyrin Hydrolysis (TBU Activity)

This method describes the procedure to determine the activity of an enzyme in TBU units per gram of enzyme (TBU/g): To a 100 mL double-walled stirred reaction vessel (kept at 40 °C) were added 20 mL of 25 mM Pi buffer at pH 7.3 and 400 μL of TBU (2%) and the emulsion stirred for 2 min. Then an automatic titrator (Metrohm) using 0.1 M sodium hydroxide was switched on, and the pH was adjusted to 7.5. Immobilized enzyme (∼30 units maximum) was added, and after the first 100 μL, base was added and the base consumption was monitored for 5 min. Unit definition: 1 TBU unit = 1 μmol butyric acid released per min/g enzyme.

#### Laurate Esterification with 1-Propanol (PLU Activity)

This method describes the procedure to determine the synthetic activity of enzyme in propyl laurate units per gram of enzyme (PLU/g): For each sample the following substrate composition was used: 200 mg (1 mmol) of lauric acid, 90 μL (1.2 mmol) of 1-propanol and 8 μL water. A shaker cell is thermostated at 60 °C. Lauric acid was weighed into the vial, and 98 μL of the 1-propanol-water mixture was added. Then about 5 mg of immobilized enzyme was weighed on a paper and added to a vial, closed, and put in the heated shaker cell. After 30 s the lauric acid melted and the timer started. After 5 min, 1.5 mL of ethanol was added, and after mixing, the resulting solution was pipetted into a 50 mL stirred beaker without the immobilized enzyme. The remaining immobilized enzyme was washed twice with 2 mL of ethanol, which was also added to the 50 mL beaker, without the catalyst. Next 50 μL of a 1% solution of thymolphthalein in ethanol was added to the solution and titrated with 0.1 M NaOH until the solution turned blue. Then 10 mL of water was added and titration continued until the solution turned blue again and the pH reached 10.5, after which the final titer was read. The acid value of 200 mg of substrate was also determined separately (blank value).
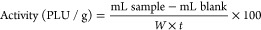
4where *W* represents the weight of enzyme in grams, *t* represents time (5 min), mL sample represents the titrated volume in mL with enzymes and mL blank the titrated volume in mL without enzymes.

#### MCT Hydrolysis in a SpinChem Rotating Bed Reactor

Wet 1,6 DAB (800 mg) was loaded into the SpinChem reactor rotor. The reactor vessel was heated to 40 °C, and then 300 mL of DI water and 30 mL of 100 mM phosphate buffer pH 7.3 were added to the SpinChem reactor. The pH was adjusted to 7.5 using 1 M NaOH, and the reaction was started by adding 0.5 mL of MCT oil. The conversion rate was followed by recording the consumption of NaOH every 5 min for 1 h. 100% conversion equals full conversion into glycerol, generating 3 equiv of free fatty acids. After each cycle, the reactor was emptied via a bottom valve, and the rotor was spun at 1000 rpm to drain the liquid from the beads. About six cycles were run in a day, and after the final 1 h cycle of the day, the enzyme was kept in the reaction mixture overnight at room temperature without stirring or titration and only emptied just prior to the next day’s cycle.

#### Cellulose Bead Enzymatic Degradation

Aliquots of beads were incubated with 5 mL of Celluclast from *T. reesei* (5 vol %) in citrate buffer (50 mM, pH 5.0) at 37 °C for 24 h. Cellulose enzymatic hydrolysis was followed by measuring the release of soluble reducing sugars in the digestion buffer. Reducing sugars were quantified through colorimetric assay with 3,5-dinitrosalicylic acid (DNS method).^51^ Aliquots (50 μL) from the digestion buffer were withdrawn, mixed with 50 μL of DNS reagent (1:1 ratio), and incubated at 90 °C for 5 min before measuring absorption (575 nm) using a FLUOstar Omega Microplate Reader—BMG LABTECH. To prepare the DNS reagent, 3,5-dinitrosalicylic acid (10 g) was mixed with DI water (≈500 mL), phenol (2 g), sodium hydroxide (10 g), and sodium sulfite (0.5 g), then additional DI water was added to reach a total volume of 1 L. To extrapolate the quantity of reducing sugars, a calibration curve was prepared using glucose as a standard.

## Supporting Information Available

Images of unmodified and diaminated cellulose beads DAB (wet and dry) and their diameter, respectively. The activity of TLL immobilized on DAB in both aqueous (dark blue bars) and organic environments (light blue bars). Periodate oxidation reaction kinetics. DO values and standard deviation are reported. The specific activity of free enzymes prior to immobilization. Retained activity (hydrolysis of *p*-nitro phenyl butyrate) upon recycling of immobilized enzymes (PDF)
