# Supplementary material for: Diaminated Cellulose Beads as a Sustainable Support for Industrially Relevant Lipases
Source: ACS Sustain Chem Eng. 2024 May 8;12(20):7703–12. doi: 10.1021/acssuschemeng.3c07849 (PMC11110057; doi:10.1021/acssuschemeng.3c07849)
Supplement: Supplementary file 1 — sc3c07849_si_001.pdf [file sc3c07849_si_001.pdf]

Diaminated cellulose beads as a sustainable support for industrially relevant lipases

Davide Califano,[a] Rob Schoevaart,[b] Katie E. Bernard,[a] Ciarán Callaghan,[c] Davide Mattia,[c] and Karen J. Edler[d]**

a. Naturbeads LTD, 2 Tetbury Hill, Malmesbury SN16 9JW, United Kingdom.

b. ChiralVision, 44 Hoog-Harnasch, 2635 DL, Den Hoorn, The Netherlands

c. Department of Chemical Engineering, University of Bath, BA27AY, UK.

d. Department of Chemistry, University of Bath, BA27AY, UK.

Number of pages: 6

Number of Figures: 4 (Figures S1-S4)

Number of Tables: 1 (Table S1)

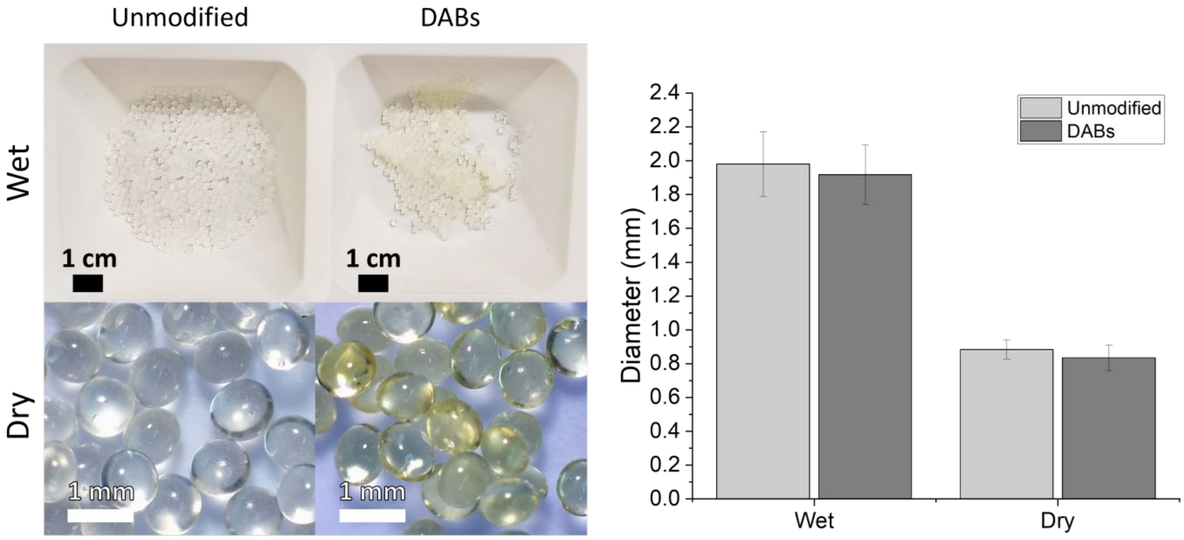

Figure S1. Images of diaminated cellulose beads (wet and dry) and their diameter. Unmodified beads exhibited a with translucent aspect while diaminated cellulose beads (DAB) turned to a pale-yellow color after reductive amination reaction. The size of both wet and dry beads did not significantly change after reductive amination.

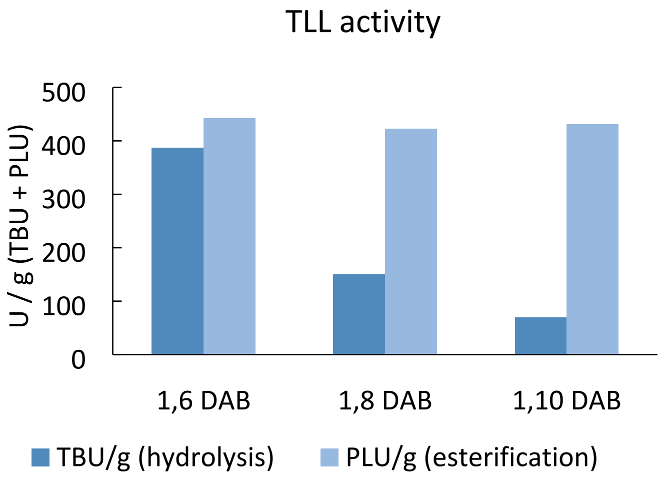

Figure S2. The activity of TLL immobilized on DAB in both aqueous (dark blue bars) and organic environments (light blue bars). In an aqueous environment (hydrolysis of tributyrin), the TLL activity dramatically decreases as the DAB carbon chain length increases while it remains constant in an organic environment (direct esterification of propyl laurate).

Table S1. Periodate oxidation reaction kinetics. In the table, the exact degree of oxidation values and standard deviation are reported.

sample code ($NaIO_4$-10)	Reaction time (hours)	$NaIO_4$/cellulose ratio(mol/mol)	degree of oxi dation (%)	standard deviation
DAC-2	2	0.23	6.84	0.72
DAC-4	4	0.23	11.44	0.63
DAC-8	8	0.23	15.13	0.81
DAC-24	24	0.23	23.51	0.18
DAC-28	28	0.23	23.01	0.31

sample code ($NaIO_4$-8)	Reaction time (hours)	$NaIO_4$/cellulose ratio(mol/mol)	degree of oxi dation (%)	standard deviation
DAC-2.5	2.5	0.18	4.78	0.29
DAC-5	5	0.18	9.51	0.03
DAC-6.5	6.5	0.18	10.71	0.26
DAC-24	24	0.18	18.83	0.09

sample code ($NaIO_4$-4)	Reaction time (hours)	$NaIO_4$/cellulose ratio(mol/mol)	degree of oxi dation (%)	standard deviation
DAC-2	2	0.09	3.38	0.045
DAC-4	4	0.09	5.27	0.85
DAC-8	8	0.09	8.24	0.76
DAC-24	24	0.09	14.95	0.27
DAC-28	28	0.09	16.71	0.04

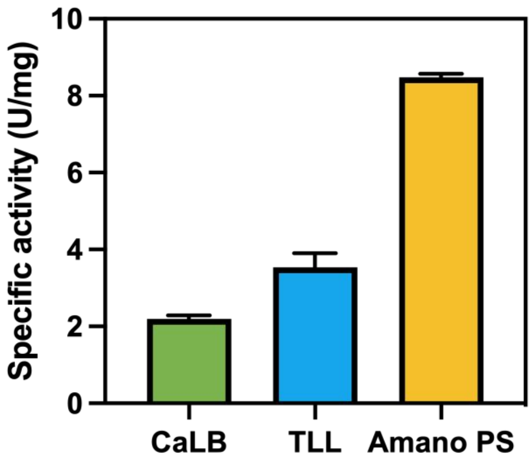

Figure S3. The specific activity of free enzymes prior to immobilization. The activity is expressed in Units per mg of enzyme (U/mg).

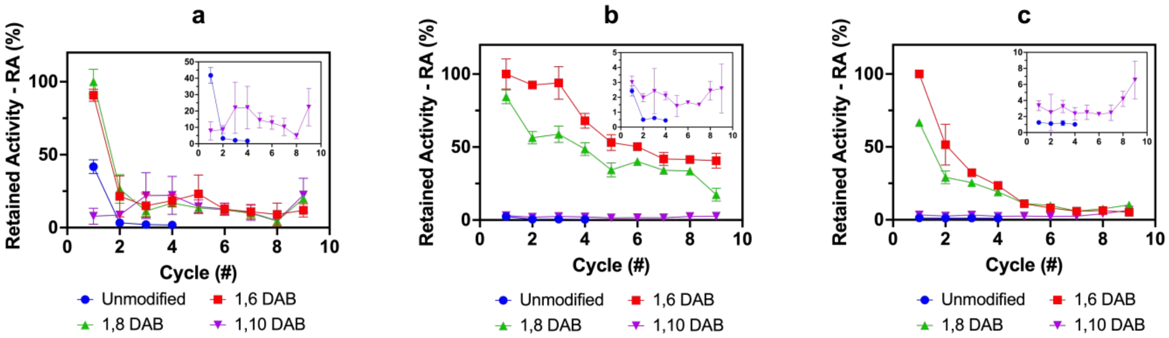

Figure S4. Retained activity (RA) (hydrolysis of *p*-nitro phenyl butyrate) upon recycling of immobilized enzymes, (a) CaLB, (b) TLL, and (c) Amano PS on 1,6 DAB, 1,8 DAB, 1,10 DAB, and unmodified beads. The retained activity is shown as the percentage of activity with respect to the highest performing sample (shown as 100% of retained activity) for each enzyme.
